# Editorial: Non-Syndromic Familial Non-Medullary Thyroid Carcinoma: Clinical and Genetic Update

**DOI:** 10.3389/fendo.2022.891903

**Published:** 2022-04-06

**Authors:** Laura Valerio, Silvia Cantara, Efisio Puxeddu, Maria Grazia Castagna

**Affiliations:** ^1^Department of Medical, Surgical and Neurological Sciences, University of Siena, Siena, Italy; ^2^Department of Medicine and Surgery, University of Perugia, Perugia, Italy

**Keywords:** thyroid cancer, papillary thyroid cancer, non medullary thyroid cancer, familial non medullary thyroid cancer, whole genome sequencing (WGS)

Non-medullary thyroid cancers (NMTCs) originate from the follicular cells of the thyroid gland and account for over 90% of all thyroid cancers. About 3-10% of NMTCs are of familial origin, and familial NMTC (FNMTC) is defined as two or more affected first-degree relatives with NMTC. Clinicopathological correlations have resulted in a further subclassification of FNMTCs into two groups. FNMTC may occur as a minor component of familial cancer syndromes (Gardner and Cowden syndrome, Carney complex type 1, Werner and DICER1 syndromes) or as a non-syndromic familial disease.

The majority of FNMTC cases are non-syndromic with unknown susceptibility gene(s). Overall, the definition, clinical behavior, and genetic mechanisms underlying FNMTC are still unclear and, thus, familial non-medullary thyroid cancer represents an interesting field of basic and clinical research.

Outstanding experts in the field contributed to the Special Research Topic titled “Non-Syndromic Familial Non-Medullary Thyroid Carcinoma: Clinical and Genetic Update”. In this Editorial, we discuss the main messages from three original research articles and one review, as reported below.

The clinical characteristics of FNMTC are controversial. Some, but not all, authors have reported an earlier age of onset, higher incidence of multifocality and lymph node metastases, and more aggressive features than sporadic thyroid cancer. Interestingly, FNMTC might be more aggressive, with higher thyroid cancer–specific mortality, in families with three or more members affected by FNMTC compared to families with “only” two members affected. Cirello et al. reported a statistically significant difference between familial papillary thyroid cancer (FPTC) and sporadic disease in terms of more aggressive presentation at diagnosis. In particular, in FPTCs, histological variants, other than the classical one, were more represented (19% vs 10%, p< 0.0001), multifocality was significantly more frequent (56% vs 38%, p= 0.02), T3-T4 tumors were more prevalent (28% vs 8%, p= 0.0003), and had a more advanced AJCC III-IV stage at diagnosis (12% *vs* 2%, p <0.0001). However, they conclude that these more aggressive features of FPTCs did not result in a worst outcome in the presence of appropriate treatments.

The genetic basis of FNMTC is complex and heterogeneous, involving not only a monogenic, but also a polygenic mode of inheritance in some families. The pathogenesis of non-syndromic familial non-medullary carcinoma has been revised by Sánchez-Ares et al. emphasizing those aspects that may be useful in clinical and pathological diagnosis. They reviewed the main FNMTC susceptibility genes, pointing out that germline mutations in non-syndromic familial PTC impact multiple processes (i.e., proliferation, migration and/or cell survival, telomere dysfunction, tumor progression, autophagy, mitochondrial metabolism, chromosomal stability, angiogenesis, etc.), while somatic events are mainly associated with the mitogen-activated protein kinase (MAPK) signaling pathway. Among all the candidate genes, a new extremely rare germline variant, c.3607A>G (p.Y1203H) in the *DUOX2* gene, has been recently reported with a “causative role” in FNMTC. Cirello et al. aimed to validate for the first time this mutation in 33 unrelated FNMTC Italian families, previously found to be negative for two susceptibility germline variants in the HAPB2 and MAP2K5 genes. Unfortunately, the *DUOX2* p.Y1203H variant was not found in either the 74 affected or the 12 not affected family members. Using the whole-genome screening (WGS) approach, Srivastava et al. identified three potentially disease-causing germline variants in *CHEK2, TIAM1*, and *EWSR1* in an NMTC-prone family. Furthermore, they proposed a synergistic model of disease progression, as all three genes are part of cell-cycle regulation and DNA damage repair pathways, thereby suggesting a polygenic mode of inheritance of FNMTC in this family. Although *CHEK2* p.E239K has been identified in breast and prostate cancers, this is the first report on the role of this variant in NMTC.

Finally, there are limited data on the role of germline mutations in mismatch repair gene variants in FNMTC. Aswath et al. analyzed the potential clinical and molecular association between hereditary nonpolyposis colorectal cancer syndrome (HNPCC) and FNMTC. They performed a large cohort study analyzing the demographic, clinical, and pathologic data of 43 family members with FNMTC and they detected, by whole-exome sequencing, a heterozygous missense variant in the *MSH2* gene exclusively in one Caucasian family meeting the clinical criteria for FNMTC and HNPCC. This result suggests that the co-occurrence of FNMTC and HNPCC-associated tumors is a rare event and a common genetic background between the two comorbidities could not be established.

With such results, we hope to expand the current knowledge regarding clinical features and genetic factors leading to FNMTC. Despite all progresses made in the last decades, we are still at an early stage in our understanding of the genetic background of non-syndromic FNMTC. Further studies are needed to gain a better understanding of the genetic factors contributing to FNMTC susceptibility to identify aggressive cases and avoid over diagnosis to promote a personalized medicine program in NMTC patients.

## Author Contributions

MC: Critical revision of the articles and final approval of the editorial version to be published LV: Drafting the article SC: Drafting the article EP: Critical revision of the articles and final approval of the editorial version to be published. All authors contributed to the article and approved the submitted version.

## Conflict of Interest

The authors declare that the research was conducted in the absence of any commercial or financial relationships that could be construed as a potential conflict of interest.

## Publisher’s Note

All claims expressed in this article are solely those of the authors and do not necessarily represent those of their affiliated organizations, or those of the publisher, the editors and the reviewers. Any product that may be evaluated in this article, or claim that may be made by its manufacturer, is not guaranteed or endorsed by the publisher.

